# Inflammation: friend or foe of bovine reproduction?

**DOI:** 10.21451/1984-3143-AR2019-0057

**Published:** 2019-10-24

**Authors:** Sylvie Chastant, Marie Saint-Dizier

**Affiliations:** 1 Reproduction, UMR INRA/ENVT 1225, Toulouse National Veterinary School, Toulouse, France.; 2 Université de Tours, UMR85 Physiologie de la Reproduction et des Comportements, Centre INRA Val-de-Loire, Nouzilly, France.

**Keywords:** inflammation, ovulation, post partum, cytokines, neutrophils

## Abstract

Inflammation is not only the first line of defense of the organism but is also required in many reproductive processes such as ovulation, corpus luteum development, luteolysis, uterine clearance after insemination and post partum. Nevertheless, if excessive or persistent, inflammation can switch from a positive mechanism to a deleterious process, impairing oocyte quality and embryo development. Not only uterine but also non genital inflammatory sites can depreciate reproductive performances, with a carry over effect of 2 to 4 months. Since the metabolic challenges of the peripartum transition period make difficult for the cow to control inflammation, dairy cows are frequently in a pro-inflammatory stage, suggesting that inflammation, rather than infection, is a limiting factor of fertility in modern dairy cows. Within the first week after calving, cows have to mount an intense inflammatory response to the bacterial invasion of the uterine cavity with the challenge of being able to switch it off in no more than 5-6 weeks. The absence of neutrophils on endometrial smear is associated with the highest success rate at insemination. Since a fine tuning – rather than an absence - of inflammation is required along the reproductive cycle, anti-inflammatory drugs do not allow any improvement of pregnancy rate, except in the specific case of embryo transfer. Appropriate management of the transition period (especially nutritional) and in a long term perspective, genetic selection contribute to improve the aptitude of cows to controls the intensity of inflammatory process.

## Introduction

(Bacterial) infection has been long considered as an essential component of reproductive disorders, whereas (sterile) inflammation is nowadays identified as a major and frequent limiting factor of reproductive performances. In the medical approach, inflammation, hallmark of “–itis” diseases, is classically considered as a deleterious process, an unwanted response leading to immune dysfunction, diversion of nutrients from productive purposes, tissue damage, sepsis, organ failure and even death. Nevertheless, from a biological perspective, inflammation, involving chemokines and cytokines release, blood vessel dilation and immune cell infiltration, is the first line immune response of an organism facing a microbial infection or a tissue injury. Since the female genital tract is physiologically exposed to a range of tissue injuries (such as ovulation) and intrauterine bacterial challenges (after calving, at insemination/mating through sperm), inflammation also belongs to the physiology of reproduction. Moreover, some other reproductive processes, such as corpus luteum development and demise, or maternal recognition of pregnancy share some similarities with inflammatory events. The objective of this paper is to review the positive and negative relationships between inflammation and cow reproduction, to finally question the rationale of the use of anti-inflammatory drugs to improve reproductive performances. This review focuses on inflammation, trying to distinguish it from the effects of bacterial infections (including Lipopolysaccharide - LPS) and on the bovine female, despite inflammation is closely associated to many physiological and pathological aspects of reproduction in many other species, if not all (e.g. Freeman *et al*., 2013 in the bitch or Katila, 2012 in the mare).

## The female genital tract is physiologically able to mount an inflammatory reaction

The female genital tract is naturally equipped to recognize pathogens and damages (Sheldon *et al*., 2018): some uterine, tubal and ovarian cells of the cow express receptors (Pattern recognition receptors, PRRs, sensors of ‘danger’) recognizing highly conserved microbial molecular signatures (MAMPs, Microbe-associated molecular patterns) or host-derived molecules indicative of cell injury (DNA fragments, mitochondrial content, but also free fatty acids and carbohydrates), referred to as DAMPs (for Damage-associated molecular patterns). Transmembrane toll-like receptors (TLRs) are probably the most classical PRRs and are expressed by bovine granulosa cells (Price and Sheldon, 2013), bovine oviductal epithelial cells, epithelial and stromal cells of the endometrium (Herath *et al*., 2009; Turner *et al*., 2014; Dadarwal *et al*., 2017; Danesh Mesgaran *et al*., 2018).

These different compartments are able to mount an early immune response: recognition of MAMPs or DAMPs by the genital cells initiate several signaling cascades (through NFκB or MAPkinase pathways for example), resulting in the expression of pro-inflammatory mediators (e.g. Tumor Necrosis Factor α -TNFα-, interleukin- IL 1 and 8), antimicrobial peptides and anti-apoptotic factors. Immune cells (mainly polymorphonuclear cells - PMN) are consequently attracted to the site of infection/injury, ensuring phagocytosis of invading microorganisms or cell fragments (Broom and Kogut 2018; Sheldon *et al*., 2019) together with the generation of *reactive oxygen species* (ROS) and the release of proteolytic enzymes. Pro-inflammatory cytokines also induce important microcirculatory events, at short (vasodilation) and long term (neoangiogenesis contributing to tissue healing).

## Physiological inflammation in reproductive processes

Apart from playing a central role into innate immunity, inflammation is essential for successful cow reproduction since inflammatory (or inflammatory-like) processes are implicated in every step of fertility: in the cycle (ovulation, corpus luteum development, luteolysis), early pregnancy (maternal recognition of pregnancy) and later, in expulsion of fetal membranes and post partum uterine involution.

### Ovulation

The ovulation exhibits many classical signs of local inflammation, with production of inflammatory mediators, locally increased blood flow, leukocyte infiltration, swelling, tissue digestion and ultimately tissue repair (Espey, 1980; Duffy *et al*., 2019). First responders to the LH surge are granulosa and theca cells, which produce chemokines and cytokines within hours after the LH surge. High concentrations of TNFα, IL1 and IL8 are found in follicular fluid at the preovulatory stage; consequently, not only the preovulatory follicle is invaded by high numbers of neutrophils and macrophages, but ovarian resident immune cells are activated (Jiemtaweeboon *et al*., 2011). Through proteolytic pathways, crucial within the ovulation process, these exogenous and endogenous cells regulate the reorganization of follicular stroma, the disruption of the granulosa basal lamina, and its invasion by vascular endothelial cells. LH-induced mediators also initiate cumulus expansion and cumulus-oocyte-complex detachment, together with extensive extracellular matrix remodeling and loss of the surface epithelium at the follicular apex. All these inflammatory phenomenons play a crucial role in the ovulatory process since treatment with antibodies directed against IL8 or neutrophils respectively suppress or decrease ovulation rate; administration of anti-proteases blocks ovulation; no blood flow increase is observed around large follicles that will finally fail to ovulate (Murdoch *et al*., 1997; Miyamoto *et al*., 2006).

### Corpus luteum development

After ovulation, the remainder of the follicle undergoes intra-antral bleeding, colonization by a large variety of immune cells (mainly macrophages, neutrophils and eosinophils), secreting numerous cytokines (TNFα, interferon gamma, interleukins, prostaglandins) together with angiogenic factors. Follicular wall is rapidly remodeled, thanks to rapid angiogenesis and granulosa/thecal cells differentiation into luteal tissue, that finally fills the former follicular antral cavity. If ovulation can be assimilated to a specific physiological injury, corpus luteum (CL) development can be compared to a phase of tissue repair and organ healing.

### Luteolysis

Not only CL formation but also lysis are inflammatory-like processes. Due to the short delay between prostaglandin F2α (PGF2α) secretion and the intraluteal immune reaction, luteolysis is even considered as an acute phenomenon (Shirasuna *et al*., 2012a). Leukocytes, especially eosinophils, macrophages and T lymphocytes, are recruited into the CL within the 5 first minutes after a PGF2α injection; as early as after 30 minutes, the expression of endothelial nitric oxide synthase is stimulated, accompanied by an increase in luteal blood flow and IL8 expression (Neuvians *et al*., 2004). Luteal blood flow increases within minutes in response to each peak of uterine PGF2α during spontaneous luteolysis in cattle (Miyamoto *et al*., 2005; Ginther and Beg, 2012). Interestingly, this “preluteolytic” blood flow increase is not observed in PGF2alpha refractory CL (Miyamoto *et al*., 2006). A little bit later, but as early as two hours, expression of pro-inflammatory cytokines (TNFα, IL1beta and interferon gamma) is increased and made responsible for apoptosis of luteal cells. CL regresses primarily through the loss of cells by apoptosis and apoptotic luteal cells are phagocytosed by macrophages. The large number of immune cells observed within the CL 6-24 hours after PGF2α are considered essential for a rapid demise of the CL tissue (Neuvians *et al*. 2004; Shirasuna *et al*., 2012b). As previously described, TNFα is also found involved into CL development: this dual effect may be due to a dose-effect, luteotropic at high doses or luteolytic at low doses, probably depending on the type of receptors activated (TNFRI or II) (Korzekwa *et al*., 2008). Four hours after PGF2α release, blood flow has felt back to the preluteolytic level and totally disappears after 24 hours (Miyamoto *et al*., 2005).

### After insemination/mating: post-mating reaction

Spermatozoa, seminal plasma or extenders are recognized as “dangers” by the genital tract and can induce an inflammatory reaction though PRRs activation. Mating or artificial insemination (AI) are thus followed by a physiological influx of neutrophils into the uterine lumen which peaks between 1 and 12 hours after. This so called post mating reaction has been observed in the uterus, cervix and vagina but not into the oviduct (despite less well studied and probably more complex). Like bacteria, sperm are phagocyted by neutrophils either directly through cell-cell attachment or entrapped with neutrophil extracellular traps (NETs) which ensnare sperm and hinder their motility (Marey *et al*., 2016). Rapid removal of sperm is thought to prevent acquired immune responses against sperm in dams since it is important for further embryo development that the female genital tract remains tolerant to paternal antigens (Katila 2012). In cattle, 60% of sperm are voided by 6 hours after AI and by 12-24 hours, only a few percent of sperm are left in the reproductive tract, the majority found within the vagina (Mitchell *et al*., 1985; Hawk, 1987). The duration of PMN infiltration is short, with a peak at less than 2 hours or at around 8-16 hours post AI or mating in cattle according to the different studies (reviewed by Katila, 2012): sperm and bacteria are rapidly eliminated, afterward the endometrium rapidly returns to a non-inflamed status, prepared to receive the embryo after its oviductal transit. If one can easily conceive than an excessive or persistent post mating response could decrease embryo survival rate, Kaufmann *et al*. (2009) suggested that the absence of post mating reaction in cows (no leukocytes intrauterine mobilization 4 hours after insemination) is associated with decreased pregnancy rates.

The situation is different in the oviduct whose epithelial cells face two opposite challenges: first, the protection against bacteria ascending from the uterus (and especially in oestrus, due to the opening of the cervical barrier and eventually insemination) and second, to favor fertilization and embryo development, whereas sperm and embryos are (semi) allogeneic to the maternal host. Interestingly, in presence of LH and estradiol, the oviduct generates a state of immunotolerance that ensures sperm survival until fertilization (Marey *et al*., 2016). Once sperm bound to oviductal epithelial cells, these cells are stimulated to secrete high levels of PGE2 that strongly suppress the PMN phagocytic activity to sperm and pro-inflammatory cytokines synthesis. Sperm binding thus favors the development of an anti-inflammatory immune environment and suppresses PMN sperm phagocytosis. More precisely, follicular fluid collected from pre-ovulatory follicle enhanced sperm phagocytosis by neutrophils in vitro whereas the oviductal fluid suppressed this activity. The oviductal environment seems thus to minimize the inflammatory effect of the follicular fluid released at the time of ovulation to allow sperm capacitation and fertilization (Yousef et al., 2019).

### Placental expulsion

Placental maturation leading to fetal membranes expulsion also involves inflammatory mechanisms, mainly protease activity and leukocytes chemotaxis (Beagley *et al*., 2010). During the third trimester of pregnancy, fetal major histocompatibility complex (MHC) Class 1 molecules begin to be expressed by placental cells and initiate a maternal response (the fetus being an allograft) (Davies *et al*., 2000). Leukocytes are recruited through the placenta via several chemoattracting cytokines (TNFα, IL2 and IL 8) and phagocyte placental cells (Heuwieser and Grunert, 1987; Kimura *et al*, 2002). In addition, Matrix MetalloProteinase and collagenase activities increase in the maternal and the fetal part of the placenta (Maj and Kankhofer, 1997; Beagley *et al*., 2010). Both inflammatory components (leukocytes and enzymes) contribute to the loosening and subsequently the detachment of the villi. Importance of efficient inflammatory processes into placental expulsion in the cow is well demonstrated by the overexpression of anti-inflammatory associated genes and decreased expression of promoters of proteolytic activity in case of spontaneous placental retention (Nelli *et al*., 2019) even if not systematically reported (Walter and Boos, 2001).

### Post partum uterine involution

Following the delivery of the calf, the uterine lumen, fulfilled with cellular and tissular debris, from placental and maternal origin, is physiologically colonized by bacteria (Sheldon *et al*., 2006). Both damages and bacterial invasion elicit a massive immediate cellular influx, whose intensity affects reproductive performances. Cows able to mount an early inflammatory response with more than 35-40% of neutrophils on endometrial smears 7 days after calving have shorter intervals from calving to pregnancy (Gilbert and Santos 2016; Cheong *et al*., 2017). This may be attributable to an early clearance of the uterine cavity from inflammatory stimuli. Inflammation is thus beneficial for the animal in the very early times after calving. However, it is important to distinguish local intrauterine cell mobilization - associated with a higher probability of ovulation from the first dominant follicle - and systemic inflammation, evaluated through haptoglobin concentration, conversely associated with a decreased ovulation rate (Cheong *et al*., 2017).

## Excessive or persistent uterine inflammation

**Figure 1:**
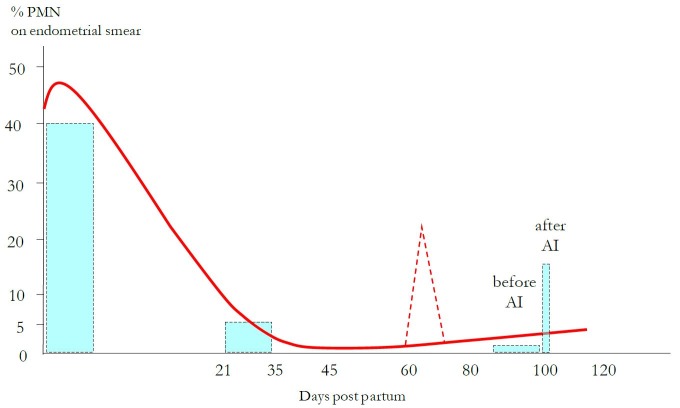
Intensity of endometrial inflammation from calving to insemination (% neutrophils on endometrial smear). All the thresholds indicated were determined based on a significant decrease of pregnancy rate. After an early intense mobilization of neutrophils after calving (>40%), inflammation is down regulated, becoming null around 40-45 days after calving and remaining null at the time of insemination. Between the nadir and the time of insemination, inflammation can be reactivated (interrupted lines). During a few hours after insemination, inflammation is transiently reactivated (post mating response).

Once the initial danger of post-partum microbial invasion is contained, it is important that inflammation is resolved, otherwise chronic inflammation persists to the detriment of tissue function. Optimal reproductive performances thus require that the animal is able to mount a rapid, acute inflammatory response to control in a short term delay the microbial invasion. In a second step, after pathogen clearance, it is of equal importance that the animal is able to control the inflammation itself, to extinct it through a timely transition to an anti-inflammatory state, favorable to tissue repair processes. Rapid, targeted, effective and quick resolution are the hallmarks of a desired inflammatory response (Broom and Kogut 2018). Considering uterine health, after the intense PMN mobilization of the first week after calving, optimal reproductive performances are obtained if the percentage of PMN on endometrial smears falls below 5% between 21 and 35 days after calving, reaching a nadir (0-1%) around 45 days after calving and being maintained at this almost null level until the time of insemination (Deguillaume, 2010; Drillich *et al*., 2012; Bogado Pascottini *et al*., 2016; Machado Pfeifer *et al*., 2018; Fig. 1).

**Figure 2:**
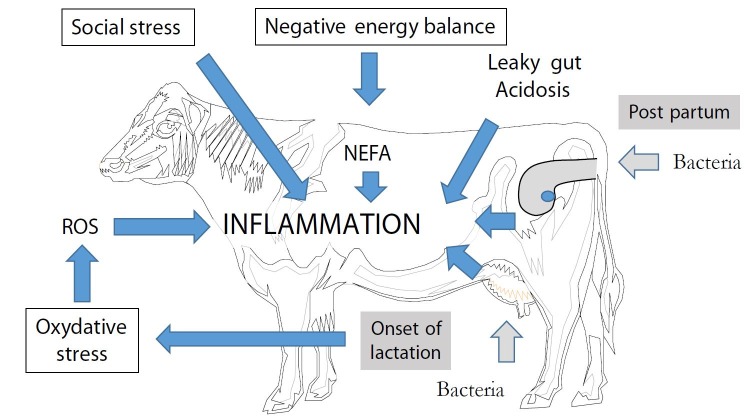
Determinants of the pro-inflammatory status during the post partum period of dairy cows. NEFA: Non Esterified Fatty Acids.

But this fine tuning of uterine inflammation (massive during the first week after calving, rapidly controlled and finally extinct at the end of the first month and transiently reactivated during a few hours after insemination) is a difficult exercise for dairy cows, due to the delicate metabolic context of the post partum period (LeBlanc, 2014). Inflammation control is not just a passive extinction but rather requires the activation of anti-inflammatory pathways (including for example lipoxins and resolvins, Sheldon *et al*., 2017). Genital health relies on a fragile equilibrium between pro- and anti-inflammatory systems, difficult to maintain in dairy cows: the persistence of uterine inflammation at the time of insemination is a frequent situation (28 to 57% of Holstein cows according to the different studies). From three weeks before and until three weeks after calving (transition period), dairy cows are facing a negative energy balance (with production of non esterified fatty acids), oxidative stress (ROS production), together with digestive acidosis and social stress (Fig. 2), all situations that put the cow in a pro-inflammatory situation. Moreover, a vicious circle installs due to the huge energy expenditure associated to the inflammatory phenomenon itself. Dairy cows use more than 1 kg glucose in the first 12 hours after an LPS challenge (Kvidera *et al*., 2017), an expenditure corresponding to about 100 kcal/kg BW^0.75^ (calculation from Gilbert, 2019), i.e. almost equivalent to maintenance. The depletion of the key cellular nutrients (such as glucose) reduces inflammatory responses, compromising the ability of animals to respond sufficiently to pathogens, resulting in the persistence of infections and chronic inflammation.

The tendency to an overactivity of pro-inflammatory systems and the instability of inflammation control in post partum dairy cows are pictured in endometrial smears follow-up: even when cows solved their uterine inflammation at 40-45 days post partum (0% PMN), transient episodes of reactivation of the uterine inflammation (up to 40% PMN) were observed after 60 days post partum (unpublished data). This explains why cows diagnosed as free from endometritis around 30 days post partum can be found with purulent uterine content at the time of insemination, probably due to a disruption of the equilibrium between pro- and anti-inflammatory systems.

To date, the unability of cows to down regulate inflammation is probably one important limiting factor of modern dairy cows fertility, due to the frequency of excessive uterine inflammation at the time of insemination, and its dramatic impact on insemination success rate (around 15 points decrease). As developed by Sheldon *et al*. (2019), uterine health is rather dependent on the endometrial tolerance to pathogens (ability to limit the disease severity induced by pathogens) than on its resistance (ability to limit the pathogen development).

## Deleterious effects of inflammation on reproduction

**Figure 3:**
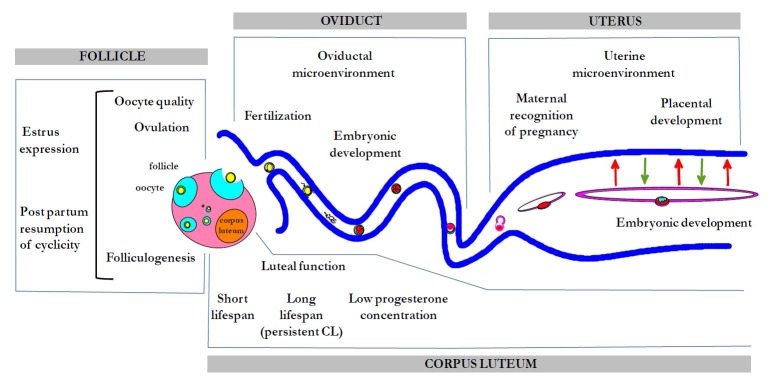
Steps of the reproductive process sensitive to inflammation.

Excessive or persistent inflammation has deleterious impact on fertility. But this applies not only to uterine inflammation, but also to extragenital inflammation. Due to cytokine release into the general circulation, ovaries, uterus and embryos may be somewhat “contaminated” by distant inflammatory sites, such as mastitis, podal inflammation, digestive inflammation consecutive to acidosis, all highly prevalent in dairy cows. Inflammatory diseases affect many steps of the reproductive process: GnRH and LH synthesis, folliculogenesis, follicular steroidogenesis, oocyte quality, ovulation, estrus expression, corpus luteum quality and lifespan, fertilization, embryo development and survival (Ribeiro and Carvalho, 2017; Fig. 3).

### Ovarian reserve

In humans and mouse, chronic inflammation is made responsible for destruction and/or premature activation of primordial follicles, leading to a decrease of the ovarian reserve, and thus Premature Ovarian Failure (phenomenon so called “inflamm-aging”) (Huang *et al*., 2019). In the bovine, considering the post partum period as a prolonged period of inflammation with excessive oxidative stress and fatty acids release, Gilbert (2019) estimated plausible that inflammatory damages could be inflicted on developing oocytes and the resting oocyte pool, resulting in chronically diminished fertility (Sheldon *et al*., 2017).

### Anovulation – Follicular cyst

Several anovulatory situations are associated with an increased expression of pro-inflammatory cytokines in the granulosa (IL1α, IL6 and TNF α) in humans (Luteinized unruptured follicle syndrom; Polycystic ovary syndrom) and in the cow (ovulation failure and follicular persistence, follicular cyst; Baravalle *et al*., 2015; Stassi *et al*., 2017).

### Oocyte competence

Inflammation mediates changes in follicular fluid that diminish the ability of the oocyte to complete meiosis, undergo fertilization and support development of a conceptus. By the activation of granulosa PRRs, steroidogenesis and the interaction between oocyte and cumulus can be impaired (Herath *et al*., 2007). Inflammatory mediators have been also described to result into aberrant spindle formation and meiosis abnormalities (Bromfield and Sheldon, 2011; Banerjee *et al*., 2012).

### Luteal insufficiency

Since inflammation affects granulosa and thecal cell function (before ovulation) and luteal cells (after ovulation), it is associated with inadequate function of the CL and insufficient circulating concentrations of progesterone, one of the major causes of infertility of modern cows (Diskin *et al*., 2011; Ribeiro *et al*., 2016).

### Embryo/placental development

Inflammation may affect embryo survival both by its deleterious effect on oocyte quality and CL function but also by providing an inadequate uterine microenvironment and through direct effect of cytokines on embryonic/placental cells. The direct influence of inflammation *per se* on embryo has been elegantly demonstrated by Hill and Gilbert (2008) who induced a non infectious endometrial inflammation; after culture into the conditioned uterine medium, blastocyst cell number was decreased, affecting trophectoderm but not inner cell mass. Other authors observed consistently impaired elongation and decreased interferon tau secretion. Inflammation thus interferes with maternal recognition of pregnancy and later, if pregnancy is maintained, decreases placental weight from Day 42 of gestation (Lucy *et al*., 2016; Ribeiro *et al*., 2016). Interestingly, maternal inflammatory diseases even caused inflammation-like changes in the transcriptome of conceptus cells (Ribeiro *et al*., 2016).

Inflammation is thus involved into many reproductive diseases, namely abnormalities in ovarian resumption of cyclicity (delayed ovulation, short luteal phases, persistent corpus luteum), metritis/endometritis and repeat breeder syndrome.

## Carry over effects of inflammation

The variety of targets sensitive to inflammation (oocyte, embryo, placenta) explains that inflammation affects reproductive performances at various distances from insemination. For example, mastitis negatively impacts on reproductive performances whatever it occurred before the first AI (even during the first month after calving), between first AI and conception or after conception, with a period at higher risk extending from 3 weeks before AI until 30 days after (Loeffler *et al*., 1999; Perrin *et al*., 2007; Lavon *et al*., 2011; Albaaj *et al*., 2017). Same observation was made with long lasting consequences of metritis on ovarian function, long after the resolution of the disease (Piersanti *et al*., 2019). This delayed effect of inflammation is reminiscent of what is known as the “Britt hypothesis” explaining the carry-over effect of negative energy balance on fertility (Britt, 1992). The carryover effect of inflammatory diseases on reproduction is attributable to the impact on oocyte quality together with an durably modified uterine environment. In case of uterine disease, inflammation can persist during several months as inflammatory lymphocytic foci within the endometrial wall, even during pregnancy (Lucy *et al*., 2016). The uterus may also be long-lasting impaired secondary to altered steroid synthesis. When previously diseased cows (retained fetal membranes, metritis, mastitis, lameness, and respiratory and digestive problems) are used as embryo recipients, establishment of diagnosed pregnancy is reduced and pregnancy loss rate is increased relative to that of previously healthy cows. The effect of inflammation on reproduction extends long beyond the resolution of the disease, until 4 months later (Ribeiro *et al*., 2016).

Transgenerational (epigenetic) effects of maternal inflammation are also suspected but with controversial observations. For Ribeiro and Carvalho (2017), female calves born from multidiseased cows have significantly lower incidence of mortality and morbidity before their first calving. Conversely, Ling *et al*. (2018) described that calves born to cows with a higher serum haptoglobin concentration (acute phase protein) during late gestation showed a lower TNFα plasma concentration after challenge, suggesting a compromised immune response to microbials.

## Suppression of inflammation: NSAID and reproduction

Since inflammation (rather than infection) is now recognized as the limiting factor of reproductive performances and in the context of the reduction of the use of antibiotics, the interest of non steroidal anti-inflammatory drugs (NSAID) has been evaluated. When used as additional treatment, NSAID allowed to limit the reproductive impact of mastitis (MacDougall *et al*., 2016). Their administration at the time of AI did not improve pregnancy rates (Heuwieser *et al*., 2011); administration before ovulation was deleterious due to an inhibition of the ovulation process and follicular cyst formation (Pugliesi *et al*., 2012). Conversely, administration at the time of embryo transfer showed an improvement of pregnancy rates (+10 to 25 points), especially when transfer was qualified as difficult (Aguiar *et al*., 2013) or after transfer of low quality embryos (Scenna *et al*., 2005). Administration at mid luteal phase targeting maternal recognition of pregnancy did not show any significant improvement of insemination success rate.

## Conclusion: Inflammation is not to be suppressed but regulated

Inflammation is a dual process, together mandatory at numerous steps of the reproduction process and deleterious for reproductive performances if excessive or persistent. Optimisation of insemination success rate depends not on the suppression of inflammation but on its fine regulation. The cow has to be able to mount intense inflammatory episodes and, more difficult, to control and shut them down rapidly, what is made complex by metabolic challenges post partum. Better regulation of the inflammation can be obtained through an appropriate dietary management during the transition period, targeting energy balance, Dietary Anions-Cations Difference, and anti oxidant reserves (LeBlanc 2012). Immunomodulators rather than anti-inflammatory drugs are an elegant strategy (such as pegbovigrastim, long acting-analog of bovine granulocyte colony-stimulating factor; Ruiz *et al*., 2017; Heiser *et al*., 2018). The genetic option is also promising, with the selection of females with high immune regulatory competences (Thompson-Crispy *et al*., 2012; Silva Silveira *et al*., 2019; König and May, 2019).
